# Fungal clones win the battle, but recombination wins the war

**DOI:** 10.1186/s43008-019-0020-8

**Published:** 2019-10-29

**Authors:** André Drenth, Alistair R. McTaggart, Brenda D. Wingfield

**Affiliations:** 1 Queensland Alliance for Agriculture and Food Innovation (QAAFI), The University of Queensland, Brisbane, QLD 4102 Australia; 2 Department of Genetics, Forestry and Agricultural Biotechnology Institute (FABI), University of Pretoria, Private Bag X20, Pretoria, Gauteng South Africa

**Keywords:** Epidemics, Evolution, Fungal adaptation, Fungal pathogens, *Fungi*, Invasive species, *Oomycota*, Sexual reproduction

## Abstract

Clonal reproduction is common in fungi and fungal-like organisms during epidemics and invasion events. The success of clonal fungi shaped systems for their classification and some pathogens are tacitly treated as asexual. We argue that genetic recombination driven by sexual reproduction must be a starting hypothesis when dealing with fungi for two reasons: (1) Clones eventually crash because they lack adaptability; and (2) fungi find a way to exchange genetic material through recombination, whether sexual, parasexual, or hybridisation. Successful clones may prevail over space and time, but they are the product of recombination and the next successful clone will inevitably appear. Fungal pathogen populations are dynamic rather than static, and they need genetic recombination to adapt to a changing environment.

## CLONES HAVE ADVANCED THE SPREAD AND SUCCESS OF FUNGI

Globalisation has increased invasions by exotic fungi that are causing major disease epidemics in new environments (Fisher et al. 2012). Epidemics of fungal pathogens in natural ecosystems and agriculture often involve founder effects whereby the invasion event is caused by a single genotype of a pathogen (Morgan et al. 2007; Rajkumar et al. 2011; Henk et al. 2012; Ordonez et al. 2015; Drees et al. 2017).

The advance of fungi to new geographic ranges over the last century, in what we term “anthropogenic exchange”, was highly successful often due to prolific clonal reproduction by invading fungi. Effective invasive pathogens infect, colonise, reproduce and establish a population following an incursion (McDonald and Linde 2002; Gladieux et al. 2015). Most fungal species encountering a new environment need to produce large numbers of spores that invade and disperse to increase the probability of successful establishment, and this favours polycyclic fungi with an asexual stage producing large numbers of spores (Horsfall 1972; McDonald and Linde 2002; Gladieux et al. 2015; Taylor et al. 2015). Highly invasive fungi in naïve environments are often well-adapted to accelerate their spread by the “Bridgehead effect” (Lombaert et al. 2010).

Invasions often involve significant genetic bottlenecks, whereby small populations or clones establish and cause disease in new environments. Founder effects combined with the ability of fungi to reproduce clonally have played a role in the common assumption that populations of many fungal pathogens are clonal. The lack of a sexual morph was often inferred. This was reflected by fungal taxonomy, which until 2011 used a dual system of nomenclature that classified sexual and asexual morphs of the same species in separate genera.

Clonal reproduction has many advantages as large numbers of asexual propagules can be produced quickly, and genotypes can be maintained without losing successful combinations of genes (McDonald and Linde 2002). Clones can colonise, persist, spread and cause epidemics, especially on susceptible hosts grown in modern monoculture-based agriculture with limited genetic diversity (Horsfall 1972; Kohn 1995). For these reasons selection has favoured clonal reproduction in fungi and fungal-like organisms, to cause rapid increase and dispersal of a population in a temporal or geographic sense, such as during an epidemic or invasion (Taylor et al. 2015). However, without adaptability and plasticity enabled through genetic recombination, an organism is less able to cope with longer-term changes in host resistance or other environmental changes, either natural or man-made (McDonald and Linde 2002; Nieuwenhuis and James 2016).

Fungi often have complicated life-cycles that may involve different modes of reproduction. Almost all fungi have the ability to reproduce asexually (clonally) through the formation of mitotic spore stages or reproduce sexually through a process of meiotic recombination to produce meiotic spores (Nieuwenhuis and James 2016). Both spore types can act as infective propagules with uniquely different infection and epidemiological characteristics. A third type of reproduction, parasexual recombination, occurs with fusion of nuclei within the mycelium followed by mitotic recombination that produces new genotypes without formation of and special sexual structures. A fourth type of reproduction occurs when homothallic ascomycetes produce viable sexual propagules that are in effect clonal.

Taylor et al. (2015) recently discussed how many species of fungi that have long-been considered clonal can also reproduce using genetic recombination, subject to suitable environmental and genetic conditions. Nieuwenhuis and James (2016) reviewed sexual reproduction in *Ascomycota* and put forward the hypothesis that truly asexual fungi are rare. Most fungi use both clonal and sexual reproduction during different stages of their life-cycle, dependent on environmental conditions, substrate, and nutrient availability.

Our opinion presented here highlights why we must consider recombination and exchange of genetic material in the formation, invasion, and dispersal of clones. We propose that all fungi have potential to reproduce through sexual (or parasexual) recombination in their life-cycles, and this should be a starting hypothesis when new fungal pathogens are encountered.

## CLONES HAVE SHAPED DOGMA IN BIOLOGY AND TAXONOMY

Clonal stages of fungi are often the most prevalent part of the life-cycle during an invasion event, and sexual reproduction may be rare in these situations (Nieuwenhuis and James 2016). Further, fungi, which sometimes have complicated life-cycles, may be unable to complete their sexual cycle if they require an alternate host or opposite mating type absent from the invasive population in a new environment (Desprez-Loustau et al. 2007; Gladieux et al. 2015). For instance, *Puccinia striiformis* (wheat stripe rust) and *Cryphonectria parasitica* (chestnut blight) are fungi that reproduce sexually in their native range but clonally at the forefront of an invasion (Milgroom et al. 2008; Dutech et al. 2010; Ali et al. 2014).

Populations of fungi in their native range are often in a coevolved relationship; a gene-for-gene equilibrium of effector and resistance genes, and a level of tolerance to pathogen virulence means that epidemics rarely occur (Thrall and Burdon 2003), providing that the hosts are not otherwise compromised. Fungal taxa may be benign and overlooked in their native environment because there are no adverse symptoms that draw attention to them.

A recent study (McMullan et al. 2018) provided an example of how two separate introductions of *Hymenoscyphus fraxineus*, the cause of Ash Dieback in Europe, have potential to kill 95% of *Fraxinus excelsior* trees in eastern Europe, whereas the source population in Asia has little impact on its native host, *Fraxinus mandschurica*. The ongoing host-pathogen interactions in the centre of origin are important drivers to increase genetic diversity in both pathogens and their host plants. In contrast, the movement of fungi to new geographic regions where susceptible plant species are present in large-scale mono-cultural settings has time and again resulted from rapid clonal reproduction of fungi that cause disease outbreaks of epidemic proportions.

There is a perception that groups of fungi known only from their mitotic stage are clonal until proven otherwise. Organisms known only from their mitotic stage, such as *Fusarium oxysporum* f.sp. *cubense* tropical race 4 of banana (recently renamed *F. odoratissimum)* (Ordonez et al. 2015; Maryani et al. 2019), may be regarded as evidence of success without sexual reproduction. But the origin of these successful genotypes remains unanswered. Plant pathologists have little justification or funding to study the time and place of recombination events in centres of origin for fungi. Instead, plant pathologists study major plant disease problems when they arise, and which are often caused by fungi that escape their natural environment and invade new geographic areas. Epidemics and outbreaks of pathogens are studied in great detail in contrast to native fungi in their centre of origin, and this has distorted our understanding of genetic diversity in natural populations of fungi.

A now redundant entire system of classification was once founded on clonal stages of fungi. Not only were taxonomists obliged to apply two (or more) names to fungi that had an asexual (clonal stage) and a sexual stage, those that were ‘strictly clonal’ were placed in a separate phylum, the *Deuteromycota* (Crous et al. 2015). The long-felt confusion caused by a dual system of nomenclature highlights the importance that biologists at the time placed on clonal stages of fungi. But, despite hypotheses that strictly asexual fungi are rare (Taylor et al. 1999; Taylor et al. 2015; Nieuwenhuis and James 2016), the dogma that some fungi exist solely as clones persists.

A clonal dogma applied to many rust fungi when their sexual stage or life-cycle was unknown. In the last decade, at least two long-studied agriculturally important rust fungi, previously hypothesized to strictly reproduce clonally, were shown to reproduce sexually. The clonal spores of coffee rust (*Hemileia vastatrix*) were thought the only viable stage until Carvalho et al. (2011) demonstrated cryptic sexual reproduction. Jin et al. (2010) resolved the life cycle of *Puccinia striiformis*, the cause of wheat stripe rust, and ended a long-held belief that it was clonal.

## SAMPLING DURING EPIDEMICS IS BIASED TOWARD CLONES

Fungal pathogens of human skin, dermatophytes, are considered clonal (Gräser et al. 2006; Persinoti et al. 2018). An example is *Trichophyton rubrum*, which had one dominant mating type and little evidence of recombination in a global population, and was hypothesised to have lost sexual reproduction when it specialised on humans (Persinoti et al. 2018). Fungal pathogens of mammals are hypothesised to use clonal reproduction to avoid production of antigenic sexual spores that may trigger an immune response (Nielsen and Heitman 2007; Wallen and Perlin 2018). However, these fungi have kept the genes required for sexual reproduction (Nielsen and Heitman 2007), and parasexual reproduction has provided a loophole for recombination without production of antigenic spores in *Candida albicans* (Forche et al. 2008).

Taylor et al. (2015) used the example of *Batrachochytrium dendrobatidis* to discuss sampling bias and context. Areas or hosts during outbreaks are heavily sampled, whereas genotypic and genetic diversity at the centre of origin, where higher levels of diversity are expected, may be overlooked because there are fewer symptoms. Host specific fungal pathogens undergo population booms, where new or invasive genotypes overcome resistance in host plants (Barrett et al. 2008); such booms are also a source of clonal sampling bias. Biogeography, sampling time, sampling technique, and host diversity will influence the effective size of a sampled population. Populations sampled from disease outbreaks or susceptible genotypes may be biased toward clonal reproduction.

Are worldwide clones of skin pathogens examples of sampling bias from successful pandemic genotypes, or are they strictly clonal fungi? Recent research (Ropars et al. 2018) showed populations of *C. albicans* that reproduced sexually had higher levels of fitness, in terms of growth rate, when compared to clonally reproducing ones, and possibly sets a precedent for recombination in other pathogens of human skin hypothesized as clonal.

## CLONES EVENTUALLY CRASH

Despite the obvious advantages of clonal reproduction, in evolutionary terms it is a short-term survival mechanism, ideally suited for rapid population expansion. Clonally reproducing pathogens encounter genetic bottlenecks giving rise to small isolated fungal populations susceptible to Muller’s Ratchet (Felsenstein 1974). Small populations also lack adaptability to changes in their environment, such as suitable combinations of effector genes required to overcome natural or introduced host plant resistance (Burdon and Silk 1997).

That clones crash is evidenced by successful control of diseases that restrict fungi to asexual reproduction. Epidemics of wheat stem rust (*Puccinia graminis*) and white pine blister rust (*Cronartium ribicola*) were controlled by removal of their alternate hosts to interrupt sexual life-cycles and stop recombination (Roelfs 1982; Maloy 1997). The management of *Zymoseptoria tritici*, the cause of wheat blotch, was improved significantly based on a realization of sexual reproduction in the life-cycle (McDonald and Mundt 2016). The sexual stage of *Z. tritici* was removed by tillage of wheat stubble, which reduced the effective population size and the advantages of sexual reproduction (McDonald and Mundt 2016).

Whether, when, where, and how recombination occurs in invasive populations should be among the first questions addressed in studies on disease epidemiology. These examples of inhibiting sexual reproduction illustrate that knowledge of genetic recombination and disease cycles are important for management of disease.

## FUNGI EXCHANGE GENETIC MATERIAL

Although sexual reproduction is costly (Lee et al. 2010), its long-term benefits outweigh those of asexual reproduction. Fungi may use a combination of asexual and sexual reproduction when possible, as demonstrated by *Phytophthora infestans*, causing late blight of potatoes. The A1 clonal mating type initially spread from the Americas to Europe in 1845, from where it became distributed pan globally as one of the earliest demonstrations of the bridgehead effect. A second invasion event of both A1 and A2 mating types in Europe rapidly displaced the previous clonal genotype with a sexually reproducing population in the 1980s (Fry et al. 1992). Sexual reproduction increased the adaptability of the pathogen and provided a new life-cycle stage, oospores, to survive in the absence of host plant material between seasons (Drenth et al. 1994).

The role of sexual reproduction in plant pathogenic fungi is often not identified, or fully realized, if long-held assumptions of life-cycles are maintained without experimentation. For example, *Austropuccinia psidii*, a rust on *Myrtaceae*, was reported to reproduce clonally on introduced *Eucalyptus* and *Syzygium* plants in its centre of diversity, Brazil, with observed genotypic diversity considered a product of mutation (Graça et al. 2013). The genetic diversity of this rust fungus on its native hosts is unknown. It was only recently shown that *A. psidii* reproduces sexually and that it produces recombinant infectious basidiospores able to cause disease (McTaggart et al. 2018). The reproductive strategies of invasive fungi, their ability to outcross (or hybridize with closely related species), and whether they form resilient spore stages to survive harsh environments, should always be primary research questions.

Fungal populations exist as temporal and spatial pools of DNA, and there are many mechanisms to acquire and share favourable genes and maintain genetic diversity (Mehrabi et al. 2017; Taylor et al. 2017). From a genetic point of view, an invasion involves the movement of genes and genomes. It is important to understand the reproductive biology of pathogens and how populations acquire genes and increase genetic diversity, including through horizontal gene transfer and various forms of hybridisation. A growing list of new pathogens are hypothesized to have evolved from interspecific hybridization between endemic and invading fungi (Newcombe et al. 2000; Brasier and Kirk 2009; Menardo et al. 2016; Stukenbrock 2016). Invasive pathogens that form hybrids will bring new challenges for disease management and highlight the importance of a detailed understanding of the disease cycle.

A fungal hybrid is a product of sexual (or parasexual) recombination between non-conspecific parents (Steenkamp et al. 2018). Fungal hybrids have been demonstrated by phylogenetic incongruence between loci (Cruywagen et al. 2017), different copies of orthologous genes within one taxon (Morin et al. 2009) and coalescence of gene regions (Stukenbrock et al. 2012). However, these approaches do not specifically test for recombination and an agreed method to identify recent F1 fungal hybrids is wanting, especially in fungi that have multiple nuclei in one cell (e.g. Basidiomycota, Oomycota). For instance, a test for hybridisation by multiple copies of orthologous genes is unable to distinguish true hybrids from heterokaryons.

## HORIZONTAL GENE TRANSFER IS NO SUBSTITUTE FOR SEXUAL REPRODUCTION IN FUNGI

Horizontal gene transfer is hypothesized to have shaped fungal biology (Fitzpatrick 2012; Feurtey and Stukenbrock 2018). For instance, fungi have acquired prokaryotic genes for metabolism through horizontal transfer (Hall et al. 2005; Marcet-Houben and Gabaldón 2010), and genes that encode compounds in some psychedelic mushrooms have been shared across genera (Reynolds et al. 2018). Horizontal gene transfer may occur without recombination and is a bona fide way to acquire novel genes and increase genetic diversity without sexual reproduction.

Entire chromosomes with suites of pathogenicity genes have been shared between species of *Fusarium* and have changed benign species into pathogens (Ma et al. 2010; van Dam et al. 2017). Conversely, entire chromosomes that lack core genes have been lost by fungi, possibly as a means to adapt (Plaumann et al. 2018). Plasticity of fungal genomes has contributed to their success, however, the question needs to be asked whether asexual fungi exclusively acquire beneficial genes from beyond normal species boundaries without sexual reproduction.

The rate of horizontal transfer in prokaryotes is high, and the potential gain from beneficial genes is hypothesised to outweigh the risk of disadvantageous transfers (Vos et al. 2015). The rate of horizontal gene transfer in eukaryotes such as fungi is expected to be lower as safeguards such as a nuclear membrane, transcription factors and differential intron processing, stop entry and introgression of foreign genetic material (Keeling and Palmer 2008; Fitzpatrick 2012). In addition, there is a knowledge gap to whether horizontal transfer of genes or whole chromosomes lead to stable introgression in the host genome in the absence of selection for the phenotype transferred.

The process of horizontal transfer between fungi has been observed experimentally (Ma et al. 2010), but it is unknown how and at what frequency such transfers occur in vivo. If horizontal transfer occurs with heterokaryosis, there is a fine line between detection of horizontal gene transfer and hybridisation. Feurtey and Stukenbrock (2018) hypothesised horizontal gene transfers will be more frequently discovered with genomic data and outlined the caveats when identifying transfers.

## THERE ARE NO STRICTLY CLONAL FUNGI

We have outlined that the prevalence of clonal stages in invading fungi misled knowledge of their full life-cycles and shaped a now superseded system of classification. Clonal reproduction is considered a good strategy for short-term survival and population expansion, while the occurrence of sexual reproduction is more suitable for long-term survival and adaptability to an ever-changing environment.

When new pathogens are discovered and appear to solely use clonal reproduction, the starting hypothesis should be that recombination is possible through sexual or parasexual reproduction, or hybridisation. We need to consider that successful clones are the product of sexual recombination and that new genotypes from recombination and hybridisation will continue challenging efforts to control plant diseases. It has long been known in agriculture that we are not dealing with static clones causing our plant disease problems but with “*Shifty* little enemies that destroy our food crops” (Stakman 1947).

## Data Availability

Not applicable.
